# Left Atrioventricular Coupling Index to Predict Incident Heart Failure: The Multi-Ethnic Study of Atherosclerosis

**DOI:** 10.3389/fcvm.2021.704611

**Published:** 2021-09-01

**Authors:** Theo Pezel, Bharath Ambale Venkatesh, Yoko Kato, Henrique Doria De Vasconcellos, Susan R. Heckbert, Colin O. Wu, Wendy S. Post, David A. Bluemke, Alain Cohen-Solal, Patrick Henry, João A. C. Lima

**Affiliations:** ^1^Division of Cardiology, Johns Hopkins Hospital, Johns Hopkins University, School of Medicine, Baltimore, MD, United States; ^2^Department of Cardiology, Lariboisiere Hospital – Assistance Publique des Hôpitaux de Paris (APHP), Inserm UMRS 942, University of Paris, Paris, France; ^3^Department of Epidemiology, University of Washington, Seattle, WA, United States; ^4^Division of Intramural Research, National Heart Lung and Blood Institute, Bethesda, MD, United States; ^5^Department of Radiology, University of Wisconsin School of Medicine and Public Health, Madison, WI, United States

**Keywords:** heart failure, cardiac magnetic resonance image, coupling, prognosis, left atria, left ventricle, multi-ethnic study of atherosclerosis

## Abstract

**Background:** Although left atrial (LA) and left ventricular (LV) structural and functional parameters have independent prognostic value as predictors of heart failure (HF), the close physiological relationship between the LA and LV suggest that the assessment of LA/LV coupling could better reflect left atrioventricular dysfunction and be a better predictor of HF.

**Aim:** We investigated the prognostic value of a left atrioventricular coupling index (LACI), measured by cardiovascular magnetic resonance (CMR), as well as change in LACI to predict incident HF in the Multi-Ethnic Study of Atherosclerosis (MESA).

**Materials and Methods:** In the MESA, 2,250 study participants, free of clinically recognized HF and cardiovascular disease (CVD) at baseline, had LACI assessed by CMR imaging at baseline (Exam 1, 2000–2002), and 10 years later (Exam 5, 2010–2012). Left atrioventricular coupling index was defined as the ratio of LA to LV end-diastolic volumes. Univariable and multivariable Cox proportional hazard models were used to evaluate the associations of LACI and average annualized change in LACI (ΔLACI) with incident HF after adjustment for traditional MESA-HF risk factors. The incremental risk prediction was calculated using C-statistic, categorical net reclassification index (NRI) and integrative discrimination index (IDI).

**Results:** Among the 2,250 participants (mean age 59.3 ± 9.3 years and 47.6% male participants), 50 incident HF events occurred over 6.8 ± 1.3 years after the second CMR exam. After adjustment, greater LACI and ΔLACI were independently associated with HF (adjusted HR 1.44, 95% CI [1.25–1.66] and adjusted HR 1.55, 95% CI [1.30–1.85], respectively; both *p* < 0.0001). Adjusted models for LACI showed significant improvement in model discrimination and reclassification compared to currently used HF risk score model for predicting HF incidence (C-statistic: 0.81 vs. 0.77; NRI = 0.411; IDI = 0.043). After adjustment, ΔLACI showed also significant improvement in model discrimination compared to the multivariable model with traditional MESA-HF risk factors for predicting incident HF (C-statistic: 0.82 vs. 0.77; NRI = 0.491; IDI = 0.058).

**Conclusions:** In a multi-ethnic population, atrioventricular coupling (LACI), and coupling change (ΔLACI) are independently associated with incident HF. Both have incremental prognostic value for predicting HF events over traditional HF risk factors.

## Introduction

Heart failure (HF) is a leading cause of mortality worldwide and a major public health issue especially in older individuals (1). The prevalence of HF is approximately 1–2% of the adult population in developed countries, rising to ≥10% among people >70 years of age (1, 2). Given the important medico-economic burden associated with HF, the American College of Cardiology/American Heart Association (ACC/AHA) guidelines reclassified HF to include stage A which includes individuals with risk factors but no structural heart disease (3). Therefore, early detection of these high-risk individuals is imperative for primary prevention. To address the need for early detection of individuals at risk for HF, several studies have assessed left atrial (LA) and left ventricular (LV) structure and function by cardiovascular magnetic resonance (CMR) (4). Several LV structural and functional parameters, such as left ventricular ejection fraction (LVEF), LV mass index, LV mass to volume ratio (LVMVR), or LV global function index (LVGFI) have shown prognostic value in predicting the occurrence of HF (5–8). However, many studies emphasize the fact that HF does not occur exclusively because of impaired LV structure and function (9, 10). Left atrial structural and functional parameters, such as LA volumes and peak LA reservoir strain have been established as an independent predictors of HF (9, 11, 12). Therefore, even with preserved LV systolic function, LA dysfunction may impair global heart performance and uncoupling between functional performance of the two chambers can also contribute to cardiac dysfunction and HF (13). These findings suggest that the LA parameters could allow earlier detection of HF risk than LV parameters. Interestingly, a study using speckle-tracking by echocardiography recently suggested a potential interest of a global atrioventricular strain in asymptomatic individuals with subclinical heart dysfunction beyond the isolated use of the LA or LV strain (14). In line with these findings, although LV and LA parameters have independent prognostic values for predicting HF, the inherently connected physiological relationship between the LA and the LV (15, 16) suggests that the assessment of left atrioventricular coupling alterations could better reflect left heart dysfunction (17). Indeed, our working group has recently demonstrated the prognostic value of a novel left atrioventricular coupling index (LACI), defined by the ratio of the LA end-diastolic volume divided by the LV end-diastolic volume by CMR, the increase of which is independently associated with cardiovascular events in MESA (18).

Previous studies have also shown the superiority of longitudinal evaluations of change in LA and LV parameters to predict HF (19–21). Therefore, we theorized that longitudinal assessment of atrioventricular coupling could be complementary to the cross-sectional evaluation to stratify the risk of incident HF among healthy individuals. Based on this rationale, we designed an analysis to examine the associations of the LACI and change in LACI with incident HF in a prospective population study of individuals without a history of clinical heart disease at baseline. Specifically, we aim to investigate the prognostic value of LACI and the average annualized change in LACI (ΔLACI) measured by CMR, for predicting incident HF in the Multi-Ethnic Study of Atherosclerosis (MESA).

## Materials and Methods

### Study Population

The MESA is a prospective, population-based multi-ethnic (White, African American, Chinese, and Hispanic) cohort study of subclinical cardiovascular disease (CVD). The details of the study design was previously described (22). In summary, between 2000 and 2002 (Exam 1), 6,814 men and women aged from 45 to 84 years, free of clinical CVD at enrollment, were recruited from six US field centers (Baltimore, MD; Chicago, IL; Forsyth County, NC; Los Angeles County, CA; Northern Manhattan, NY; and St Paul, MN). Exam 1 was followed by Exam 2 (2002–2004), Exam 3 (2004–2005), Exam 4 (2005–2007), and Exam 5 (2010–2012). Participants with cardiovascular risk factors were not excluded. Participants with any significant valvular disease (stenosis or regurgitation) at baseline were excluded. The methodology of baseline characteristics and outcome collection is detailed in Supplementary File 1. All participants provided written informed consent. All study protocols were approved by the institutional review boards of each participating field center.

**Figure 1:**
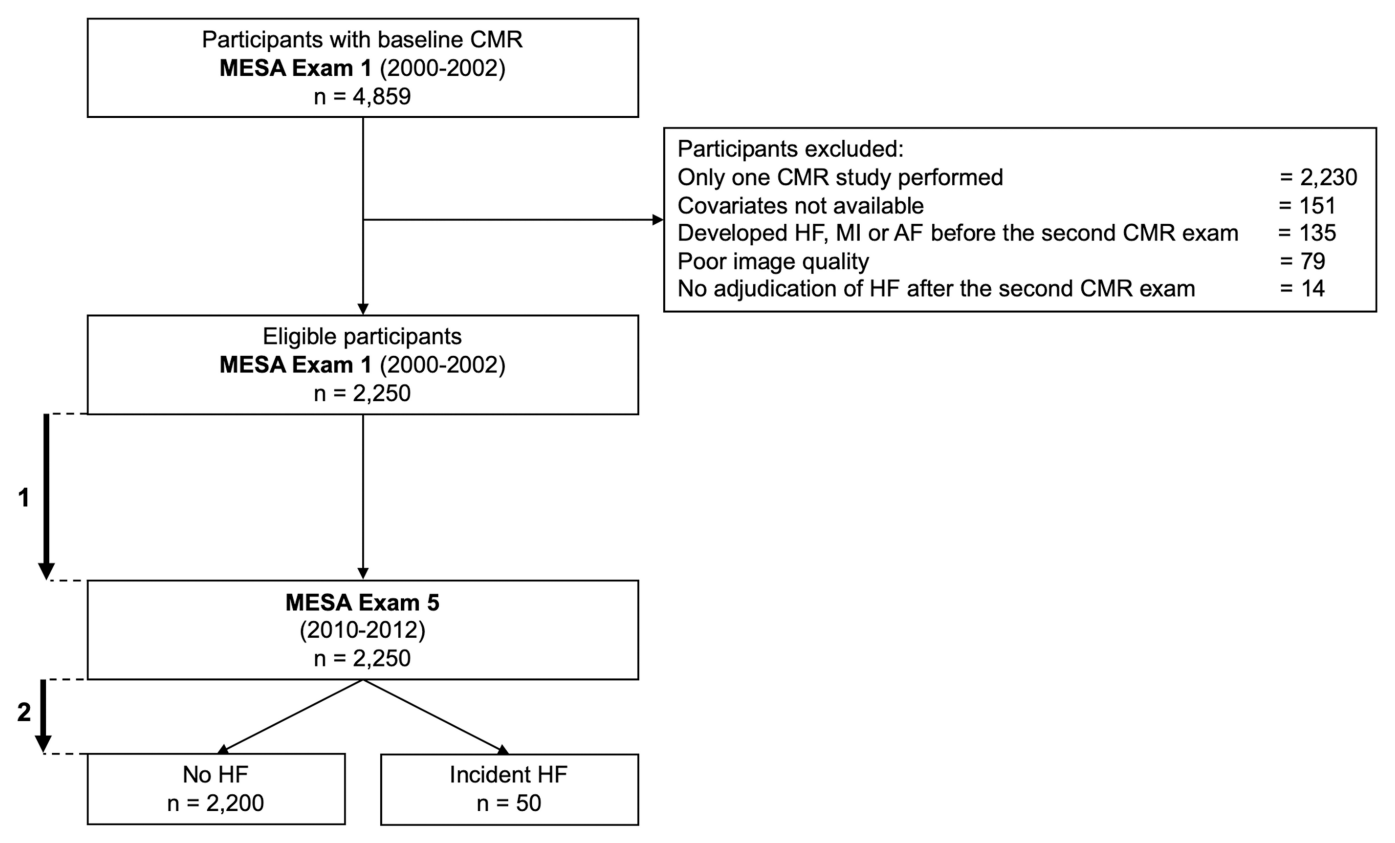
Flowchart of the study. (1) Mean time between baseline and second CMR exams: 9.6 ± 0.6 years. (2). Mean time of HF follow-up: 6.8 ± 1.3 years after the second CMR exam. AF, atrial fibrillation; CMR, cardiovascular magnetic resonance; HF, heart failure; MI, myocardial infarction.

A flowchart of the MESA population investigated in the current study is depicted in Figure 1. Participants were excluded if: (i) they did not have the second CMR exam, (ii) their images were missing or not of sufficient quality to allow measurement of LA and LV volumes, or (iii) they developed incident HF, myocardial infarction, or atrial fibrillation, including patients who had HF during AF, between Exam 1 and Exam 5 (Figure 1). Of note, incident HF between Exam 1 and Exam 5 was defined as any episode of acute HF irrespective of its etiology, including acute HF secondary to other cardiac conditions. Of the 4,859 participants with baseline CMR that included LA volume assessment (Exam 1), 2,250 participants returned for a second CMR exam at Exam 5 after a mean time of 9.6 ± 0.6 years and were included in the study.

### CMR Protocol and Image Analysis

Cardiovascular magnetic resonance was performed with 1.5 T MR scanners, either Signa LX or CVi (GE Medical Systems, Waukesha, WI, USA) or Symphony or Sonata (Siemens Medical Systems, Erlangen, Germany). Long-axis cine images were obtained from 2-chamber and 4-chamber views, using electrocardiogram-gated fast gradient-echo pulse sequences. A stack of short-axis cine images was acquired to encompass both ventricles, and LV end-diastolic volume was measured using cardiac image modeler software (CIM version 6.0, University of Auckland, New Zealand). All the cine images were acquired with a temporal resolution of ~50 ms. The complete CMR protocol, as well as details on image analysis, data quality control, calculations for LVEF, LV mass and volumes, LA volumes, and measurement reproducibility, have been published previously (23).

Multimodality tissue tracking software (MTT version 6.0, Toshiba Medical Systems Corporation, Tokyo, Japan) was used to quantify LA volume and strain from 2- and 4-chamber cine CMR images (Supplementary File 2). This method has been validated previously with good to excellent intra- and inter-reader reproducibility with intraclass correlation (ICC) of 0.88 to 0.98 (*p* < 0.001), and good inter-study reproducibility with ICC of 0.44 to 0.82 (*p* < 0.05 to 0.001) (24–26). A single experienced operator, blinded to the participant's case status, defined endocardial and epicardial borders of the LA at end-systole. Using the marked points, the software creates endocardial and epicardial borders, then tracks LA tissue in subsequent frames. The endocardial and epicardial contours generated by the software are then followed by the operator during the cardiac cycle for quality control.

### Left Atrioventricular Coupling Index

**Figure 2:**
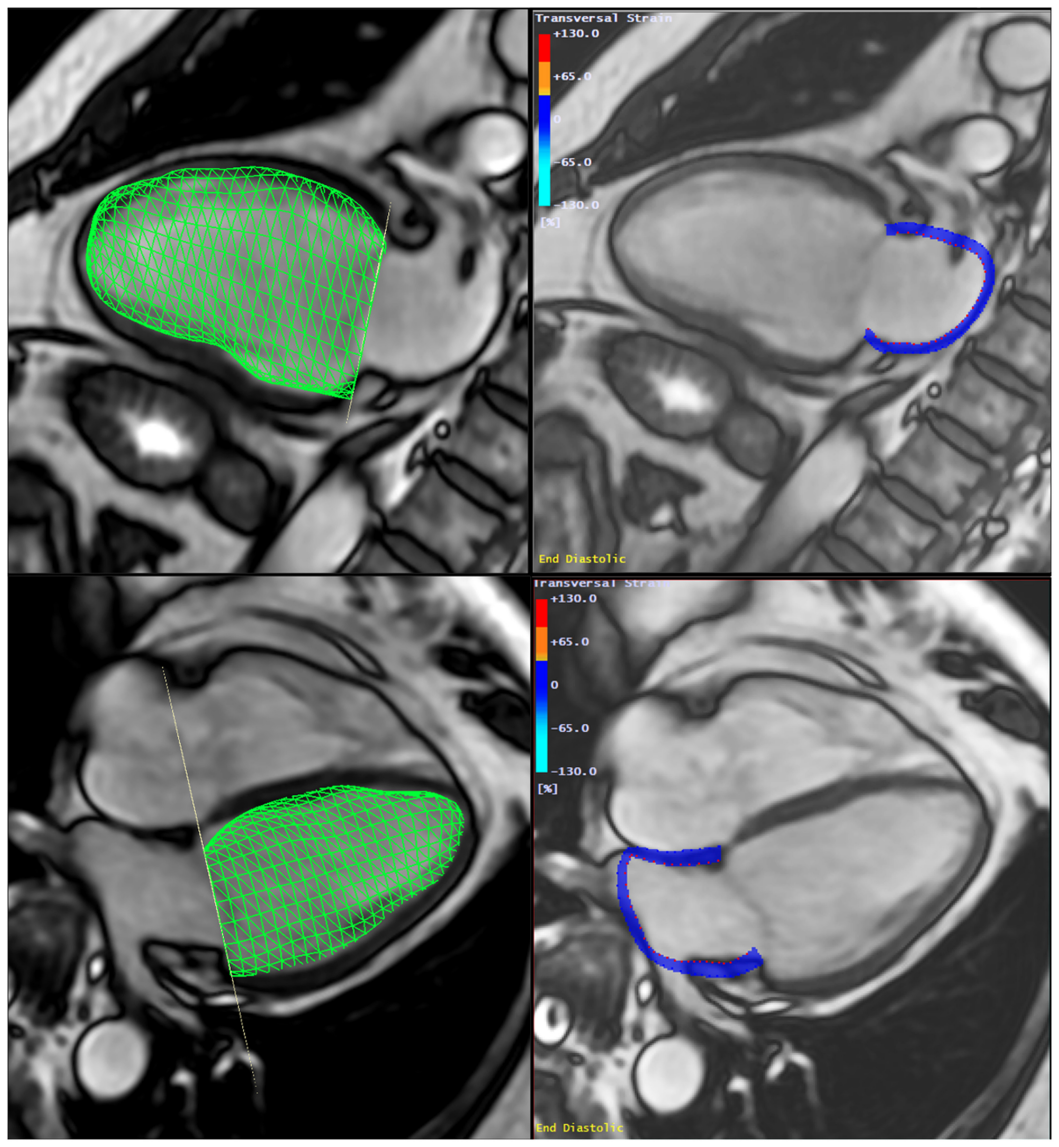
Method to assess the Left Atrio-ventricular Coupling Index (LACI) by CMR. The LACI was defined by the ratio between the LA end-diastolic volume and the LV end-diastolic volume. A stack of short-axis cine images was acquired to encompass both ventricles and LV end-diastolic volume was measured using cardiac image modeler (CIM) software (green volume, left panel). LA end-diastolic volume was measured using multimodality tissue-tracking (MTT) software to track LA wall motion during the end-diastole in the 4-chamber and 2-chamber views (pink borders, right panel). CMR, cardiovascular magnetic resonance; LA, left atrial; LACI, left atrioventricular coupling index; LV, left ventricle.

The LACI was defined by CMR for each participant by the LA end-diastolic volume divided by the LV end-diastolic volume. The LV volume was measured from the stack of short-axis cine images, while the LA volume was measured from the 2-chamber and 4-chamber views, as previously described (Figure 2). The LA and LV volumes were measured in the same end-diastolic phase defined by mitral valve closure.

The LACI value is expressed as a percentage, and a higher LACI indicates greater disproportion between the LA and LF volumes at ventricular end-diastole, reflecting greater impairment of left atrioventricular coupling. Moreover, the ΔLACI is defined by the annual difference in the LACI value measured at baseline, at Exam 1 (LACI_Baseline_) and the LACI value measured after 10-years, at Exam 5 (LACI_10−years_), and the ΔLACI value is expressed as a percentage per year.

### Incident Heart Failure

The MESA outcome event ascertainment protocols have been described in detail and are available online (www.mesa-nhlbi.org). In addition to MESA follow-up examinations, a telephone interviewer contacted each participant (or representative) every 9–12 months to inquire about interim hospital admissions, CV outpatient diagnoses, and mortality.

Medical records were reviewed and diagnoses of HF events, including HF with preserved or reduced ejection fraction, were adjudicated by a panel of MESA physicians using standardized criteria. We used both probable and definite HF events for analysis. Probable HF was defined as a physician diagnosis and a receipt of HF medical treatment with intravenous diuretics. Definite HF required an additional criterion; such as evidence of pulmonary congestion on chest radiography, reduced LV function by echocardiography or ventriculography, or evidence of LV diastolic dysfunction. Ejection fraction (EF) measures were recorded from clinical echocardiography for events diagnosed as HF by MESA cardiac reviewers. The last HF events data was followed-up to December 2017. To avoid any competitive risk between HF events and AF, we excluded all patients experienced AF during the follow-up.

### Statistical Analyses

The baseline and after 10-year participant characteristics are presented as mean ± standard deviation (SD) for continuous variables and as counts and percentages for categorical variables in Table 1. Comparisons employed the χ^2^ or Fisher's exact test for categorical variables and the Student's *t*-test or Mann–Whitney–Wilcoxon test, as appropriate, for continuous variables. We used Cox regression models to study the associations between the LACI, or ΔLACI, and incident HF events. The assumption of proportionality of hazards was confirmed for each model. The cumulative risk of incident HF over the follow-up years for the cohort, stratified by the LACI terciles, or ΔLACI terciles, was determined using Kaplan–Meier curves, censored at the most recent follow-up. Differences across terciles were compared using the log-rank test.

**Table 1:** Population characteristics of participants at baseline and at second examination (*n* = 2,250).

**Parameters**	**Baseline (Exam 1)** **(*n* = 2,250)**	**Second study (Exam 5), 9.6 ± 0.6 years after baseline**		
		**No HF (*n* = 2,200)**	**HF (*n* = 50)**	***p*-Values**
Age, years	59.3 ± 9.3	68.6 ± 9.1	76.0 ± 8.9	**<0.001**
Male, *n* (%)	1,050 (46.7)	1,026 (46.6)	24 (48.0)	0.962
Ethnicity (Ca/Ch/AA/Hi), %	43/122/24/21	43/12/24/21	25/1/12/12	0.171
Hypertension, *n* (%)	840 (37.3)	1,226 (55.7)	45 (90.0)	**<0.001**
Systolic blood pressure, mmHg	123 ± 20	123 ± 20	135 ± 24	**0.001**
Diastolic blood pressure, mmHg	72 ± 10	68 ± 10	69 ± 11	0.839
Hypertension medication, *n* (%)	701 (31.2)	1,130 (51.4)	40 (80.0)	**<0.001**
Body mass index, kg/m^2^	27.8 ± 5.0	28.1 ± 5.2	28.7 ± 5.3	0.399
Glycemic status, *n* (%)				**0.021**
Normal	1,781 (79.2)	1,381 (62.8)	23 (46.0)	
Impaired fasting glucose	254 (11.3)	443 (20.1)	13 (26.0)	
Diabetes mellitus	215 (9.6)	376 (15.7)	14 (28.0)	
Smoking status, *n* (%)				
Never	1,178 (52.4)	1,023 (46.5)	14 (28.0)	
Former	817 (36.3)	1,013 (46.0)	33 (66.0)	
Current	255 (11.3)	164 (7.5)	3 (6.0)	
LDL cholesterol, mg/dl	118 ± 31	107 ± 32	91 ± 32	**0.001**
HDL cholesterol, mg/dl	51 ± 15	56 ± 16	56.4 ± 18	0.877
Lipid-lowering medication, *n* (%)	331 (14.7)	811 (36.9)	24 (48.0)	0.143
NT-proBNP, pg/ml	73.6 ± 108.2	117.7 ± 122.0	463.4 ± 232.2	**<0.001**
Framingham CVD risk, %	12.3 ± 8.9	15.2 ± 9.0	20.6 ± 8.5	**<0.001**
Heart rate, bpm	62 ± 8.9	64.2 ± 10.4	66.8 ± 10.4	0.092
LA parameters				
LAVI_min_, ml/m^2^	11.9 ± 6.2	16.3 ± 8.3	26.1 ± 16.3	**<0.001**
LAVI_max_, ml/m^2^	30.0 ± 9.4	35.1 ± 11.2	43.9 ± 16.4	**<0.001**
Peak LA reservoir strain, %	37.0 ± 11.0	31.7 ± 13.7	23.9 ± 16.5	**0.002**
LV parameters				
LV EDVi, ml/m^2^	70.9 ± 12.1	64.4 ± 13.2	67.0 ± 17.3	0.295
LVEF, %	62.6 ± 5.7	62.1 ± 7.1	59.1 ± 9.16	**0.027**
LV mass index, g/m^2^	65.0 ± 11.6	65.7 ± 13.4	76.2 ± 16.3	**<0.001**
LV MVR, g/ml	0.93 ± 0.17	1.04 ± 0.22	1.20 ± 0.35	**0.005**
LVGFI, %	40.4 ± 6.1	37.6 ± 6.7	33.0 ± 7.4	**<0.001**
LACI, %	17.0 ± 8.0	26.1 ± 10.2	41.2 ± 12.1	**<0.001**

*AA, African American; Ca, Caucasian; Ch, Chinese American; Hi, Hispanic; NT-proBNP, N-terminal prohormone of brain natriuretic peptide; CVD, cardiovascular disease; HDL, high-density lipoprotein; HF, heart failure; LA, left atrium; LACI, left atrioventricular coupling index; LAVI, left atrium volume indexed; LDL, low-density lipoprotein; EDVi, end-diastolic volume indexed; LV, left ventricle; LVEF, left ventricle ejection fraction; LVGFI, LV global function index; LVMVR, LV mass/LV volume. Bolded p-values correspond to statistically significant results with p < 0.05*.

The HF risk prediction model used was the MESA-HF risk model already described (27). Two models were proposed to assess the associations between the ΔLACI, or average annualized change in all other LA and LV parameters, and incident HF. In Model 1, we adjusted for the following traditional MESA-HF risk factors (27) at the second CMR exam after 10-year (Exam 5): age, sex, race, heart rate, body mass index, hypertension, diabetes, smoking status, dyslipidemia, and N-terminal prohormone of brain natriuretic peptide (NT-proBNP). Model 2 included the model 1 plus the baseline value of the parameter assessed, measure to account for baseline differences when measuring change, and potential measurement error bias (28).

Model discrimination was assessed with Harrell's C-statistic. Incremental risk prediction was calculated using categorical net reclassification index (NRI) and integrative discrimination index (IDI) for 7-year follow-up. Risk categories for NRI were defined a priori (<5%, 5–10%, and >10%), similar to that used in other studies (27).

The survival tree method was used to determine the cut-off to transform the LACI and ΔLACI into a binary variable with the best predictive value for HF. A two-tailed *p*-value <0.05 was considered statistically significant. All data were analyzed using *R* software, version 3.6.1 (R Project for Statistical Computing).

## Results

### Study Population

Among the 4,859 MESA participants with baseline CMR studies including LA volume assessment, 2,250 (46.3%) had at least two CMR exams (baseline and after 10-year follow-up) with LA, LV, and outcome data available (mean age 59.3 ± 9.3 years and 46.7% male participants). Among those, 37.3% had hypertension with 31.2% on antihypertensive therapy, 11.3% were current smokers, 9.6% had diabetes mellitus, and the mean body mass index was 27.8 ± 5.0 kg/m^2^. The baseline characteristics of the study population at Exam 5 after a mean time of 9.6 ± 0.6 years, divided into those who developed HF or not, are presented in Table 1. Among the patients excluded due to atrial fibrillation during the follow-up, only 12 patients had incident HF during atrial fibrillation. After a mean follow-up time of 6.8 ± 1.3 years after the second CMR exam, 50 participants had incident HF events. Among these 50 incident HF events, there were 39 definite HF (78%) and 11 probable HF (22%). Of these 50 incident HF events, there were 29 HF with preserved LVEF (58%) and 21 with reduced LVEF (42%).

Participants with HF were older (*p* < 0.001) and had more frequently hypertension (*p* < 0.001) with a higher systolic blood pressure level (*p* = 0.001) compared to participants without HF. LA and LV functional parameters were lower (all *p* < 0.001), and LV mass/LV volume higher (*p* = 0.005) in participants with HF compared to those without AF.

### LACI and Annualized Change in LACI

For the entire study population, mean baseline LACI was 17.0 ± 8.0% and at follow up, LACI_10−years_ was 26.3 ± 10.5%, with a mean ΔLACI of 1.3 ± 1.0%/year (Supplementary File 3). Change in LACI (ΔLACI) and individual LA and LV parameters over 9.6 ± 0.6 years are shown in Supplementary File 4. While participants who developed HF had greater increase in LA volume (**Δ**LAVI_min_ 1.29 ± 1.28 vs. 0.47 ± 0.81 ml/m^2^/year, *p* < 0.001) than those who did not, LV end-diastolic volumes decreased similarly with aging in both groups. Of note, correlations between LA and LV end-diastolic volumes were weak at both baseline and follow up (*R*^2^ = 0.15 and R^2^ = 0.10) (Supplementary File 5).

There was no significant difference in mean LACI between women and men at baseline (LACI_Baseline_ = 16.7 ± 8.2 vs. 16.8 ± 7.6%, *p* = 0.66, respectively), but at follow up, mean LACI was higher in women than in men (LACI_10−years_ = 26.3 ± 12.0 vs. 24.7 ± 11.2%, *p* = 0.010, respectively). Consistently, **Δ**LACI was higher in women than in men (1.03 ± 1.10 vs. 0.83 ± 1.00%/year, *p* < 0.001, respectively) (Supplementary File 6).

### LACI and Incident HF

**Figure 3:**
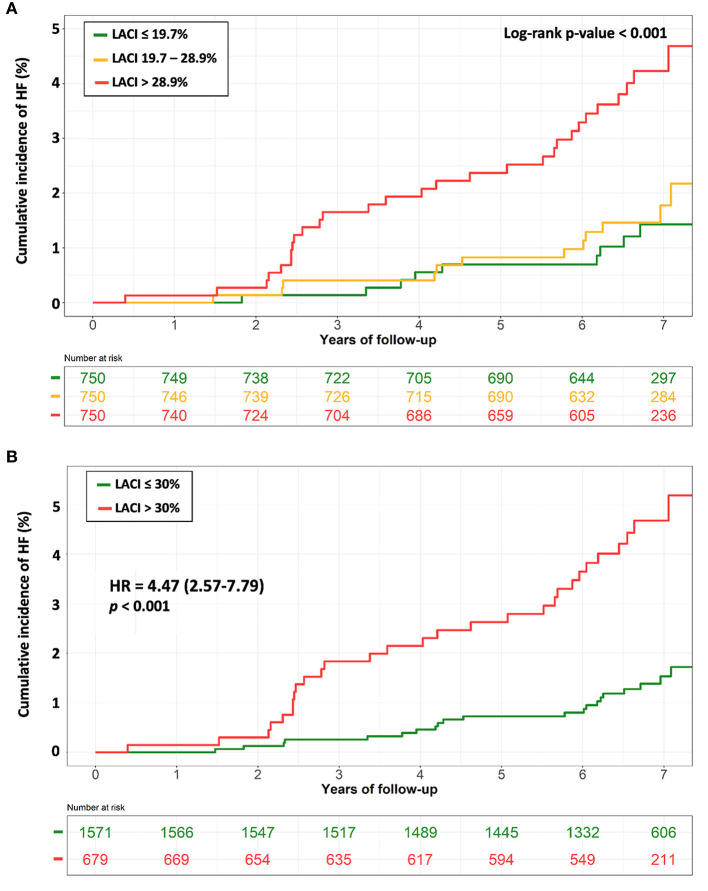
Kaplan-Meier survival curves for incident HF stratified by LACI terciles **(A)** and by a LACI cut-off of 30% **(B)**. **(A)** The cumulative hazard was significantly greater in the 3^th^ LACI_10−years_ tercile compared with the other terciles for incident HF (log-rank for difference; *p* < 0.001). **(B)** The cumulative hazard was significantly greater for patients with LACI_10−years_ >30% compared with patients with LACI_10−years_ ≤ 30% for incident HF (log-rank for difference; *p* < 0.001). HF, heart failure; LACI, left atrioventricular coupling index.

The results of unadjusted and adjusted Cox proportional hazard models for LACI as well as LA and LV parameters measured after 10-years are presented in Table 2. LACI_10−years_ was positively associated with incident HF before and after adjustment for risk factors (adjusted hazard ratio [HR] 1.44; 95% CI [1.25–1.66] per 1 SD increment; *p* < 0.001). LACI_10−years_ top tercile (LACI_10−years_ >28.9%) was more strongly associated with HF incidence than the bottom tercile (<19.7%) (log-rank *p* < 0.001) (Figure 3A). Using an optimal cut off point to predict incident HF defined by survival tree method (Supplementary File 7), LACI_10−years_ >30% was independently associated with incident HF before (HR 4.47; 95% CI [2.57–7.79], *p* < 0.001) and after adjustment (adjusted HR 2.05; 95% CI [1.14–3.68], *p* = 0.011) (Figure 3B).

**Table 2:** Univariable and multivariable analysis of incident HF according to LACI and other LA or LV parameters after 10 years.

	**Univariable analysis**		**Model 1** ^*^	
			**HF risk factors**	
	**Hazard ratio**	***p*-Values**	**Hazard ratio**	***p*-Values**
	**(95% CI)**		**(95% CI)**	
${\text{LACI}}_{10-\text{years}}^{†}$	1.69 (1.50–1.90)	**<0.001**	1.44 (1.25–1.66)	**<0.001**
LACI_10−years_ cut-off >30%^‡^	4.47 (2.57–7.79)	**<0.001**	2.05 (1.14–3.68)	**0.011**
LAVI_min_	1.67 (1.47–1.88)	**<0.001**	1.40 (1.28–1.68)	**<0.001**
LAVI_max_	1.64 (1.36–1.98)	**<0.001**	1.35 (1.08–1.69)	**0.023**
Peak LA reservoir strain	0.75 (0.58–0.88)	**0.003**	0.79 (0.65–0.92)	**0.012**
LV EDVi	1.20 (0.92–1.57)	0.174	0.95 (0.78–1.16)	0.619
LVEF	0.65 (0.50–0.85)	**0.002**	0.70 (0.55–0.89)	**0.008**
LV mass index	1.58 (1.30–1.97)	**<0.001**	1.44 (1.25–1.66)	**<0.001**
LACI_10−years_ cut-off >30%^‡^	4.47 (2.57–7.79)	**<0.001**	2.05 (1.14–3.68)	**0.011**
LAVI_min_	1.67 (1.47–1.88)	**<0.001**	1.40 (1.28–1.68)	**<0.001**
LAVI_max_	1.64 (1.36–1.98)	**<0.001**	1.35 (1.08–1.69)	**0.023**
Peak LA reservoir strain	0.75 (0.58–0.88)	**0.003**	0.79 (0.65–0.92)	**0.012**
LV EDVi	1.20 (0.92–1.57)	0.174	0.95 (0.78–1.16)	0.619
LVEF	0.65 (0.50–0.85)	**0.002**	0.70 (0.55–0.89)	**0.008**
LV mass index	1.58 (1.30–1.97)	**<0.001**	1.22 (1.03–1.52)	**0.032**
LV MVR	1.64 (1.34–2.02)	**<0.001**	1.37 (1.07–1.74)	**0.016**
LVGFI	0.49 (0.37–0.65)	**<0.001**	0.54 (0.40–0.74)	**<0.001**
Framingham CVD risk	1.84 (1.39–2.43)	**<0.001**	1.00 (0.64–1.57)	0.984
tbf <0.001	1.22 (1.03–1.52)	**0.032**
LV MVR	1.64 (1.34–2.02)	**<0.001**	1.37 (1.07–1.74)	**0.016**
LVGFI	0.49 (0.37–0.65)	**<0.001**	0.54 (0.40–0.74)	**<0.001**
Framingham CVD risk	1.84 (1.39–2.43)	**<0.001**	1.00 (0.64–1.57)	0.984

*Of note, each line of this table corresponds to the addition one by one of the LV or LA parameters to the model 1. All LV parameters, LA parameters and LACI values were normalized according to the following formula: (parameter – mean value)/standard deviation. CI, confidence interval; CVD, cardiovascular disease; EDVi, end-diastolic volume indexed; EF, emptying fractions; HF, heart failure; Indexed volumes, maximum (VImax), minimum (VImin); LA, left atrial; LACI, left atrioventricular coupling index; LAVI, left atrium volume indexed; LV, left ventricle; LVEF, left ventricle ejection fraction; LVGFI, LV global function index; MVR, mass-to-volume ratio*.

* *Multivariable **model 1** (HF risk model) included: age, gender, race, body mass index, hypertension, diabetes, smoking status dyslipidemia, and NT-proBNP*.

*^†^LACI_10−years_ used as continuous variable*.

‡ *LACI_10−years_ used as binary variable defined by a cut-off >30%. Bolded p-values correspond to statistically significant results with p < 0.05*.

### Annualized Change in LACI and Incident HF

**Figure 4:**
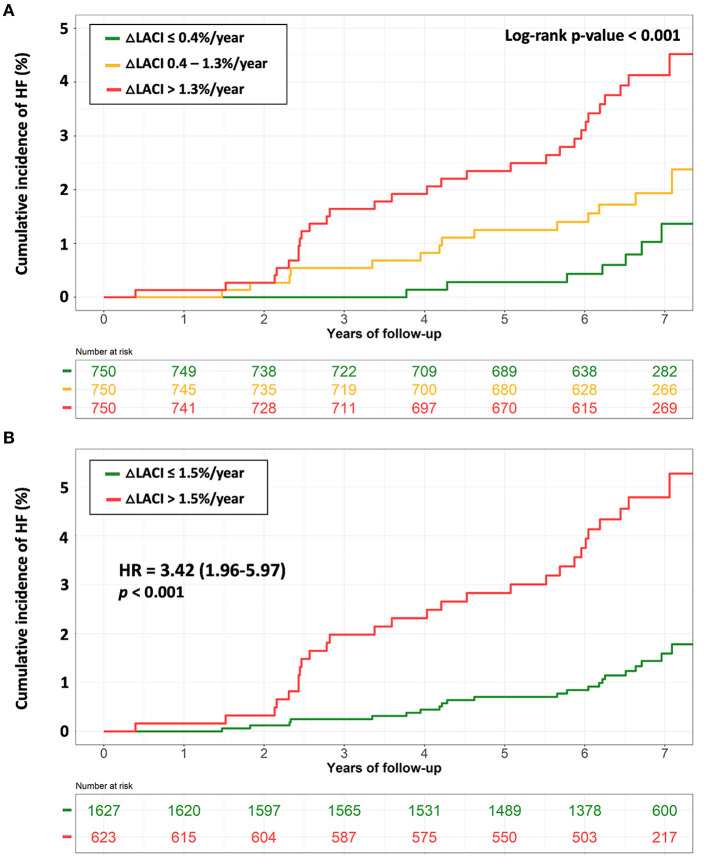
Kaplan-Meier survival curves for incident HF stratified by terciles of ΔLACI **(A)** and by ΔLACI with a cut-off of 1.5%/year **(B)**. **(A)** The cumulative hazard was significantly greater in the 3^th^ tercile compared with the other terciles for incident HF (log-rank for difference; *p* < 0.001). **(B)** The cumulative hazard was significantly greater for patients with ΔLACI >1.5%/year compared with patients with ΔLACI ≤ 1.5%/year for incident HF (log-rank for difference; *p* < 0.001). **Δ**, annual change; HF, heart failure; LACI, left atrioventricular coupling index.

Bivariable and multivariable analyses results for ΔLACI and main LA and LV parameters are presented in Table 3. Annual change in LACI was positively associated with HF after adjustment on LACI_Baseline_ (bivariable analysis), (HR 1.77; 95% CI [1.49–2.09], *p* < 0.001). After adjusting for traditional MESA-HF risk factors (Model 1) plus LACI_Baseline_ (Model 2), **Δ**LACI remained independently associated with incident HF (adjusted Model 1 HR 1.56; 95% CI [1.32–1.85] per 1 SD increment; adjusted Model 2 HR 1.55; 95% CI [1.30–1.85] per 1 SD increment; respectively, *p* < 0.001 for both). **Δ**LACI top tercile (>1.3%/year) was more strongly associated with incident HF than the bottom tercile (<0.4%/year) (log-rank *p* < 0.001) (Figure 4A).

**Table 3:** Bivariable and multivariable analysis of incident HF according to Annual change in LACI and Annual change in other LA or LV parameters.

	**Bivariable analysis** ^*^		**Model 1** ^**†**^		**Model 2** ^**‡**^	
			**HF risk factors**		**Model 1 + Baseline LA/LV variables**	
	**Hazard ratio**	***p*-Values**	**Hazard ratio**	***p*-Values**	**Hazard ratio**	***p*-Values**
	**(95% CI)**		**(95% CI)**		**(95% CI)**	
**Δ**LACI^§^	1.77 (1.49–2.09)	**<0.001**	1.56 (1.32–1.85)	**<0.001**	1.55 (1.30–1.85)	**<0.001**
**Δ**LACI cut-off>1.5%/year^||^	3.74 (2.14–6.55)	**<0.001**	2.53 (1.44–4.46)	**<0.001**	2.68 (1.51–4.75)	**<0.001**
**Δ**LAVI_min_	1.69 (1.47–1.93)	**<0.001**	1.50 (1.25–1.80)	**<0.001**	1.48 (1.22–1.79)	**<0.001**
**Δ**LAVI_max_	1.52 (1.31–2.02)	**<0.001**	1.45 (1.11–1.90)	**<0.001**	1.52 (0.97–1.62)	0.064
ΔPeak LA reservoir strain	0.72 (0.56–0.87)	**0.002**	0.88 (0.62–1.04)	0.078	0.70 (0.52–0.85)	**0.019**
**Δ**LV EDVi	1.17 (0.87–1.58)	0.293	1.15 (0.89–1.47)	0.279	1.14 (0.87–1.46)	0.291
**Δ**LVEF	0.68 (0.51–0.91)	**0.009**	0.78 (0.60–1.01)	0.055	0.67 (0.50–0.88)	**0.004**
**Δ**LV mass index	1.59 (1.39–2.10)	**<0.001**	1.27 (0.99–1.61)	0.065	1.51 (1.26–1.82)	**<0.001**
**Δ**LV MVR	1.48 (1.19–1.85)	**<0.001**	1.24 (0.98–1.57)	0.071	1.32 (1.04–1.67)	**0.020**
**Δ**LVGFI	0.51 (0.37–0.70)	**<0.001**	0.77 (0.59–1.00)	0.051	0.75 (0.56–1.05)	0.065
**Δ**Framingham CVD risk	1.20 (0.89–1.60)	0.228	1.01 (0.79–1.29)	0.920	1.02 (0.74–1.40)	0.912

*Of note, each line of this table corresponds to the addition one by one of the changes in LV or LA parameters to the models 1 or 2. All variables values were expressed per 1-SD/year and normalized according to the following formula: (Variable measured – mean value)/standard deviation. **Δ**, Annual change; CI, confidence interval; CVD, cardiovascular disease; EDVi, end-diastolic volume indexed; EF, emptying fractions; HF, heart failure; Indexed volumes, maximum (VImax), minimum (VImin); LA, left atrial; LACI, left atrioventricular coupling index; LV, left ventricle; LVEF, left ventricle ejection fraction; LVGFI, LV global function index; MVR, mass-to-volume ratio*.

* *Bivariable model included both the annual change in the variable and the value of the variable measured at baseline*.

† *Multivariable **model 1** (HF risk model) included: age, gender, race, body mass index, hypertension, diabetes, smoking status dyslipidemia and NT-proBNP*.

‡ *Multivariable **model 2** included: model 1 + baseline value measured at Exam 1 for each LA or LV parameters*.

§ ***Δ**LACI used as continuous variable*.

|| ***Δ**LACI used as binary variable defined by a cut-off >1.5%/year. Bolded p-values correspond to statistically significant results with p < 0.05*.

Using an optimal **Δ**LACI cut-off of >1.5%/year to predict incident HF defined by survival tree method (Supplementary File 8), an increase in **Δ**LACI of >1.5%/year remained independently associated with greater HF occurrence (adjusted Model 1 HR 2.53; 95% CI [1.44–4.46] per 1 SD increment; adjusted Model 2 HR 2.68; 95% CI [1.51–4.75] per 1 SD increment, respectively, *p* < 0.001 for both) (Figure 4B).

### Atrioventricular Coupling Improvement of HF Risk Prediction

The multivariable model with the LACI_10−years_ showed significant improvement in model discrimination compared to the multivariable model with traditional MESA-HF risk factors for predicting incident HF (C-statistic: 0.81 vs. 0.77; NRI = 0.411; IDI = 0.043). Follow up exam LACI_10−years_ also demonstrated better discrimination for incident HF than the multivariable model with individual LA or LV parameter plus the traditional MESA-HF risk factors (Table 4).

**Table 4:** Discrimination and reclassification associated with LACI to different LA and LV parameters at 10-years of follow-up to predict incident HF.

	**Incident HF**		
	**C-index**	**NRI**	**IDI**
	**(95%CI)**	**(95%CI)**	**(95%CI)**
Model 1^*^ (HF risk factors)	0.77 (0.73–0.82)	Reference	Reference
Model 1 + ${\text{LACI}}_{10-\text{years}}^{†}$	0.81 (0.74–0.87)	0.411 (0.042–0.780)	0.043 (0.016–0.106)
Model 1 + LACI_10−years_ cut-off >30%^‡^	0.80 (0.73–0.86)	0.607 (0.063–0.843)	0.039 (0.011–0.107)
Model 1 + LAVI_min_	0.80 (0.73–0.86)	0.201 (-0.219–0.486)	0.038 (0.010–0.104)
Model 1 + LAVI_max_	0.78 (0.74–0.82)	0.328 (0.050–0.573)	0.015 (0.004–0.041)
Model 1 + Peak LA reservoir strain	0.79 (0.73–0.85)	0.312 (0.047–0.599)	0.017 (0.006–0.044)
Model 1 + LV EDVi	0.77 (0.73–0.82)	0.075 (−0.222–0.372)	0.000 (−0.001–0.010)
Model 1 + LVEF	0.80 (0.74–0.86)	0.369 (0.158–0.580)	0.039 (0.010–0.109)
Model 1 + LV mass index	0.79 (0.72–0.85)	0.248 (0.137–0.398)	0.018 (0.009–0.067)
Model 1 + LV MVR	0.79 (0.73–0.85)	0.259 (0.143–0.402)	0.020 (0.012–0.069)
Model 1 + LVGFI	0.80 (0.74–0.85)	0.382 (0.157–0.607)	0.031 (0.015–0.085)
Model 1 + Framingham CVD risk	0.77 (0.73–0.82)	0.065 (−0.192–0.337)	0.001 (−0.001–0.012)

*All LV parameter, LA parameter and LACI values were normalized according to the following formula: (parameter–mean value)/standard deviation. For each model, discrimination and reclassification were based on net reclassification improvement (NRI) and integrated discrimination improvement (IDI). Results are for 7-year follow-up. CI, confidence interval; CVD, cardiovascular disease; EDVi, end-diastolic volume indexed; EF, emptying fractions; HF, heart failure; Indexed volumes, maximum (VImax), minimum (VImin); LA, left atrial; LACI, left atrioventricular coupling index; LV, left ventricle; LVEF, left ventricle ejection fraction; MVR, mass-to-volume ratio*.

* *Multivariable **model 1** (HF risk model) included: age, gender, race, body mass index, hypertension, diabetes, smoking status, dyslipidemia, and NT-proBNP*.

*^†^LACI_10−years_ used as continuous variable*.

‡ *LACI_10−years_ used as binary variable defined by a cut-off > 30%*.

### Improvement in Risk Prediction With Addition of Average Annualized Change in LACI

After adjustment, ΔLACI showed significant improvement in model discrimination compared to the multivariable model with traditional MESA-HF risk factors for predicting incident HF (C-statistic: 0.82 vs. 0.77; NRI = 0.491; IDI = 0.058). ΔLACI also demonstrated better discrimination for incident HF than the multivariable model with average annualized changes in LA or LV parameters (Table 5).

**Table 5:** Discrimination and reclassification associated with Annual change in LACI to change in different LA and LV parameters to predict incident HF.

	**Incident HF**		
	**C-index**	**NRI**	**IDI**
	**(95%CI)**	**(95%CI)**	**(95%CI)**
Model 1^*^ (HF risk factors)	0.77 (0.73–0.82)	Reference	Reference
Model 2^†^ + **Δ**LACI^‡^	0.82 (0.76–0.89)	0.491 (0.048–0.934)	0.058 (0.028–0.096)
Model 2^†^ + **Δ**LACI cut-off>1.5%/year^§^	0.81 (0.75–0.87)	0.536 (0.050–0.998)	0.045 (0.024–0.083)
Model 2^†^ + **Δ**LAVI_min_	0.80 (0.75–0.85)	0.455 (0.003–0.907)	0.031 (0.008–0.076)
Model 2^†^ + **Δ**LAVI_max_	0.79 (0.74–0.83)	0.270 (−0.010–0.482)	0.019 (0.002–0.072)
Model 2^†^ + **Δ**Peak LA reservoir strain	0.77 (0.73–0.82)	0.009 (−0.178–0.281)	0 (-0.002–0.007)
Model 2^†^ + **Δ**LV EDVi	0.77 (0.73–0.82)	−0.013 (−0.172–0.198)	0 (-0.002–0.009)
Model 2^†^ + **Δ**LVEF	0.77 (0.73–0.82)	0.010 (−0.182–0.278)	0 (-0.001–0.008)
Model 2^†^ + **Δ**LV mass index	0.80 (0.75–0.84)	0.428 (0.002–0.876)	0.030 (0.007–0.075)
Model 2^†^ + **Δ**LV MVR	0.79 (0.73–0.85)	0.251 (−0.030–0.532)	0.016 (0.002–0.053)
Model 2^†^ + **Δ**LVGFI	0.80 (0.74–0.86)	0.466 (0.006–0.926)	0.033 (0.011–0.080)
Model 2^†^ + **Δ** Framingham CVD risk	0.77 (0.73–0.82)	−0.052 (−0.246–0.262)	0 (−0.001–0.008)

*All variables values were expressed per 1-SD/year and normalized according to the following formula: (Variable measured – mean value)/standard deviation. For each model, discrimination and reclassification were based on net reclassification improvement (NRI) and integrated discrimination improvement (IDI). Results are for 7-year follow-up. **Δ**, Annual change; CI, confidence interval; CVD, cardiovascular disease; EDVi, end-diastolic volume indexed; EF, emptying fractions; Indexed volumes, maximum (VImax), minimum (VImin); LA, left atrial; LACI, left atrioventricular coupling index; LV, left ventricle; LVEF, left ventricle ejection fraction; MVR, mass-to-volume ratio*.

* *Multivariable **model 1** (HF risk model) included: age, gender, race, body mass index, hypertension, diabetes, smoking status dyslipidemia and NT-proBNP*.

† *Multivariable **model 2** included: model 1 + baseline value measured at Exam 1 for each LA or LV parameters*.

‡ ***Δ**LACI used as continuous variable*.

§ ***Δ**LACI used as binary variable defined by a cut-off>1.5%/year*.

## Discussion

In this multi-ethnic population of participants, aged from 45 to 84 years, and free of clinical CVD at enrollment, our findings suggest the predictive value of both a novel LACI and the average annualized change in LACI, ΔLACI, for predicting incident HF. Indeed, LACI and ΔLACI were independently associated with incident HF, improving model discrimination and reclassification beyond traditional MESA-HF risk factors. To our knowledge, the prognostic value of this index and its incremental prognostic value over and above traditional MESA-HF risk factors have not been previously reported.

In our study, LACI and ΔLACI were stronger independent predictors of incident HF than the Framingham score and individual LA or LV parameters, resulting in improved discrimination and reclassification for incident HF. The increase in LA volume relative to that of the LV at end-diastole reflects impaired LV compliance, leading to a reduction of LA reservoir function, which have been described as significant predictors of incident HF (9). Using the survival tree method, we also investigated the best LACI and ΔLACI cut-off points to predict incident HF, and found that LACI >30% and ΔLACI >1.5%/years were also independently associated with incident HF. Therefore, LACI appears to reflect an earlier stage of LA remodeling than individual LA parameters, having stronger prognostic value for predicting incident HF before and after adjustment for traditional MESA-HF risk factors. In line with previous reports (9), these findings suggest that HF may not occur exclusively because of impaired LV structure or function, but may also be susceptible to uncoupling of LA and LV structure and function as markers of early LV diastolic dysfunction. Interestingly, although the multivariable model with the peak LA reservoir strain showed significant improvement in model discrimination and reclassification compared to the multivariable model with traditional MESA-HF risk factors for predicting incident HF, this study did not show an incremental prognostic value of the annual change in peak LA reservoir strain to predict incident HF.

A previous CMR study performed in 40 healthy individuals has described that the oldest individuals had larger LA and smaller LV volumes with larger LA/LV end-diastolic volume ratio (27 ± 6 vs. 19 ± 3%; *p* < 0.001) and preserved LVEF (29). These effects of aging on left atrioventricular coupling and LV filling are consistent with our findings. Consistently, in a canine model of early-stage hypertensive HF with preserved LVEF, left atrioventricular coupling assessed by CMR was impaired and the curvilinear LA end-reservoir pressure-volume relationship was shifted upward and leftward, indicating reduced LA compliance (30). Consistently, a recent study described a LACI measured by echocardiography as a prognosticator of death in patients with HF with reduced LVEF or degenerative mitral disease and regurgitation (31). Thus, all these findings emphasize the prognostic importance of atrioventricular coupling reflected by intricate hemodynamic interactions between LA and LV during LV diastole (32).

Regarding the question of the optimal time of the cardiac cycle to assess this LACI, some reports have described the important interaction between the performance of LA and LV, in the absence of mitral valve disease, particularly at the end of LV diastole (15, 16). Furthermore, a recent study has consistently suggested that both LA end-diastolic volume (33, 34) and LA end-diastolic volume change (35, 36) are more closely correlated with LV filling pressure and the occurrence of CV events, including HF, than these same measurements measured in systole (16).

To investigate the important interaction between LA and LV performance during the LV end-diastole some studies have evaluated in detail the ventricular filling mechanism. During the LV diastole, the passive filling creates an early blood flow vortex inside the LV at the beginning of LV diastole (17). This diastolic blood flow vortex generates an important kinetic energy and redirects the incoming LA inflow toward the LV outflow tract, priming the LV by stretching cardiomyocytes and maximizing pre-load before the onset of LV systolic contraction (37). All of these mechanisms emphasize the important hemodynamic diastolic interactions between LA and LV, possibly in part explaining the prognostic value of left atrioventricular coupling measured at that moment (LACI).

Finally, early detection of a subclinical left atrioventricular coupling impairment could pave the way to new therapeutic strategies that might slow or change their clinical history, impacting on their quality of life and mortality. Further studies could be proposed, evaluating early pharmacologic effects on left atrioventricular coupling.

### Study Limitations

In this study, LACI was investigated as a diagnostic tool for early detection of HF risk in asymptomatic participants without known CVD. Because LACI may not be regarded as an ideal assessment tool for individuals with pronounced LA and LV enlargement in case of advanced structural heart disease, the extension of our findings to populations with established CVD require additional evaluation. Due to the relatively low incidence of HF, the current findings should be analyzed with precaution. However, the exclusion of all participants with significant valvular disease, myocardial infarction, or atrial fibrillation at the starting time of the time-dependent analysis reduces the risk of confounding bias. In addition, HF was not differentiated into HF with preserved or reduced LVEF, due to the limited power for sub-analysis given the low number of events. Moreover, the main cause of HF was not adjudicated in all patients. This study allowed to assess the incremental value of LACI and ΔLACI beyond traditional MESA-HF risk factors but not beyond LV parameters such as LVEF because due to a risk of collinearity in the model. Because the distribution of LACI and ΔLACI were not exactly normal, the time-dependent analyses used scaled LACI (LACI-mean value/SD) and scaled ΔLACI (ΔLACI-mean value/SD), which makes its clinical interpretation less easy. ΔLACI was averaged across ten years, thus assuming linearity over time. This method may not have fully captured the variation in year-to-year measurements thus providing additional precedence for further investigation. In this regard, the concept of dynamic change in risk profile, as participants age and accumulate exposure to risk factors, has been explored using other prediction models in cardiology, suggesting that risk profile change may be superior to single baseline assessments (38–40). We also used two instead of three dimensional methods to measure LA volumes, which may have underestimated true volumes by 11.5–20% (41). However, this method has been widely used and validated in clinical studies, being particularly suitable for population work with large sample sizes such as the present study (24, 25). Dedicated LV fibrosis parameters such as T1 mapping or late gadolinium enhancement were not available to perform specific analysis. Although the current study provides important clues to understand the HF pathophysiology and the potential role of the left atrioventricular coupling, the relatively low event rate warrants further studies to validate the prognostic value of LACI and its annual change.

## Conclusion

In a large multi-ethnic population free of clinical CVD at baseline, impaired left atrioventricular coupling reflected as greater LACI and ΔLACI measured by CMR, were associated with higher risk of incident HF during a 7-year median follow-up. The addition of LACI and ΔLACI to risk prediction models for incident HF improved model discrimination and reclassification for incident HF risk. Future studies should validate these findings to better understand the role of left atrioventricular coupling in HF pathophysiology and risk prediction.

## Data Availability Statement

The raw data supporting the conclusions of this article will be made available by the authors, without undue reservation.

## Ethics Statement

The studies involving human participants were reviewed and approved by Standard Operating Procedures (SOPs) of the IRBs. The patients/participants provided their written informed consent to participate in this study.

## Author Contributions

All authors participated in the discussion of the concept of the study. TP, BA, and JL analyzed data and drafted the manuscript with critical revision. As authors, we attest to each of our substantial contributions to the manuscript and revision. All authors have read and approved the final manuscript.

## Author Disclaimer

All authors declare that the submitted work is original and has not been published before (neither in English nor in any other language) and that the work is not under consideration for publication elsewhere. The views expressed in this manuscript are those of the authors and do not necessarily represent the views of the National Heart, Lung, and Blood Institute; the National Institutes of Health; or the U.S. Department of Health and Human Services.

## Conflict of Interest

The authors declare that the research was conducted in the absence of any commercial or financial relationships that could be construed as a potential conflict of interest.

## Publisher's Note

All claims expressed in this article are solely those of the authors and do not necessarily represent those of their affiliated organizations, or those of the publisher, the editors and the reviewers. Any product that may be evaluated in this article, or claim that may be made by its manufacturer, is not guaranteed or endorsed by the publisher.

## Abbreviations

CMR: cardiovascular magnetic resonance
CVD: cardiovascular disease
HF: heart failure
LV: left ventricle
LVEF: left ventricular ejection fraction
LVMVR: LV mass to volume ratio
LA: left atrium
LACI: left atrioventricular coupling index
LACI_Baseline_: left atrioventricular coupling index measured at baseline (MESA Exam 1)
LACI_10−years_: left atrioventricular coupling index measured after 10-years (MESA Exam 5)
ΔLACI: average annualized change in left atrioventricular coupling index
MESA: multi-ethnic study of atherosclerosis.
