# Supplementary material for: Left Atrioventricular Coupling Index to Predict Incident Heart Failure: The Multi-Ethnic Study of Atherosclerosis
Source: Front Cardiovasc Med. 2021 Sep 1;8:704611. doi: 10.3389/fcvm.2021.704611 (PMC8442844; doi:10.3389/fcvm.2021.704611)

**SUPPLEMENTARY FILES**

**Table of Contents:**

- Supplementary file 1: Methodology of baseline characteristics and outcomes collection.
- Supplementary file 2: Methodology of LA measurements by CMR.
- Supplementary file 3: Distributions of LACI and Annual change in LACI.
- Supplementary file 4: Annual change in LACI, LA and LV variables between Exam 1 and Exam 5
- Supplementary file 5: Relationship between the LA end-diastolic volume and LV end-diastolic volume used to define the LACI at Exam 5.
- Supplementary file 6: LACI and Annual change in LACI regarding the participant’s Sex
- Supplementary file 7: Determination of the optimal cut-off to transform the LACI at Exam 5 into a binary variable with the best predictive value for incident HF.
- Supplementary file 8: Determination of the optimal cut-off to transform the annual change in LACI into a binary variable with the best predictive value for incident HF.

**SUPPLEMENTARY FILE 1:**

**Methodology of baseline characteristics and outcomes collection**

***A) Baseline characteristics***

- Standardized questionnaires were used at baseline (Exam 1) and after 10 years (Exam 5) to collect information about age, sex, race/ethnic background and cigarette smoking. A medication inventory was used to collect information on prescription and nonprescription medications.
- Cigarette smoking was categorized as current, former or never. Body mass index was calculated as weight divided by height squared (kg/m^2^) with weight measured to the nearest 0.5kg and height to the nearest 0.1 cm. Blood pressure was measured 3 times using a Dinamap model Pro 100 automated oscillometric sphygmomanometer (Critikon; Tampa, FL) while the participants were resting in a seated position. The average of the last two measurements were used in the analysis. Fasting glucose was obtained by a thin-film adaptation of the glucose oxidase method (Johnson & Johnson Clinical Diagnostics, Inc, Rochester, NY). Diabetes mellitus was defined as a fasting glucose of ≥126 mg/dL or use of hypoglycemic medication.
- Blood samples were stored at −70°C and were thawed before testing (maximum of 3 freeze-thaw cycles). NT-proBNP was measured using the Elecsys 2010 system (Roche Diagnostics, Indianapolis, IN). All analyses were performed at a core laboratory (Veteran’s Affairs San Diego Healthcare System, La Jolla, CA). Intra- and interassay coefficients of variation at various concentrations of NT-proBNP were 1.3% and 4.8%, respectively (*Karl, J, et al. Development of a novel, N-terminal-proBNP (NT-proBNP) assay with a low detection limit. Scand J Clin Lab Invest Suppl. 1999;230:177–181*). The analytical measurement range for NT-proBNP was 5 to 35 000 pg/mL.

***B) Outcomes***

- The diagnosis of myocardial infarction (MI) during the follow-up period between Exam 1 and Exam 5 required either abnormal cardiac biomarkers (two times upper limits of normal) regardless of pain or ECG findings; evolving Q waves regardless of pain or biomarker findings; or a combination of chest pain, and ST-T evolution or new LBBB, and biomarker levels 1-2 times upper limits of normal.
- The diagnosis of atrial fibrillation (AF) during the follow-up period between Exam 1 and Exam 5 were identified through MESA surveillance and, for participants enrolled in fee-for-service Medicare, from inpatient and outpatient Medicare claims data. As a part of standard event surveillance procedures, all hospitalizations were identified during follow-up calls to study participants or a proxy. Discharge diagnosis and procedure codes from those hospitalizations were abstracted. AF was documented as present if an International Classification of Diseases diagnosis code for AF or atrial flutter (version 9: 427.31 or 427.32; version 10: I48) was recorded.

**SUPPLEMENTARY FILE 2:**

**Methodology of LA measurements by CMR**

Maximum, pre-atrial, and minimum contraction left atrium (LA) volumes were extracted from volume curves that were created using the area-length method from apical 2- and 4-chamber views, using the following formula for Biplane calculation:$Volume= \frac{(0.0848 \times area\text{4ch }\times area\text{4ch})}{(\left[ \mathrm{length}\text{4ch}+length\text{2ch} \right]/2)}$.

All LA volumes were indexed to body surface area (ml/m^2^). Biplanar volume and function assessment on MTT had a strong positive linear correlation and concordance to other manual methods (e.g. Simpson’s method)(23).

The software calculates global longitudinal atrial strain by averaging longitudinal strain of all LA segments in 2- and 4-chamber views during each cardiac cycle. Global peak longitudinal LA strain (peak LA strain) was measured from the global longitudinal strain curve.

The LA cycle has been described in 3 phases, a reservoir collecting pulmonary venous flow during ventricular systole, a conduit for passage of blood into the left ventricle during early diastole and a booster pump by augmenting ventricular filling during late diastole via atrial contraction.

The LA indexed volume variables were:

- Maximum (LAVImax): LA volume at end systole, before opening of mitral valve
- Minimum (LAVImin): LA volume at end diastole, after mitral valve closure

Using these measured LA volumes, the total LA emptying fractions (EF) was calculated as follows:

- Total LA EF (%) = (LAVmax−LAVmin)/LAVmax.

**SUPPLEMENTARY FILE 3:**

**Distributions of LACI and Annual Change in LACI.**

Distribution of LACI_Baseline_, measured at Exam 1 (A), LACI_10-years,_ after the second CMR exam at Exam 5 (B), Annual change in LACI between Exam 1 and Exam 5 (C) and evolution of the distribution between LACI_Baseline_ and LACI_10-years_ after the second CMR exam at Exam 5 (D). Abbreviations: CMR: cardiovascular magnetic resonance; LACI: left atrioventricular coupling index.

**
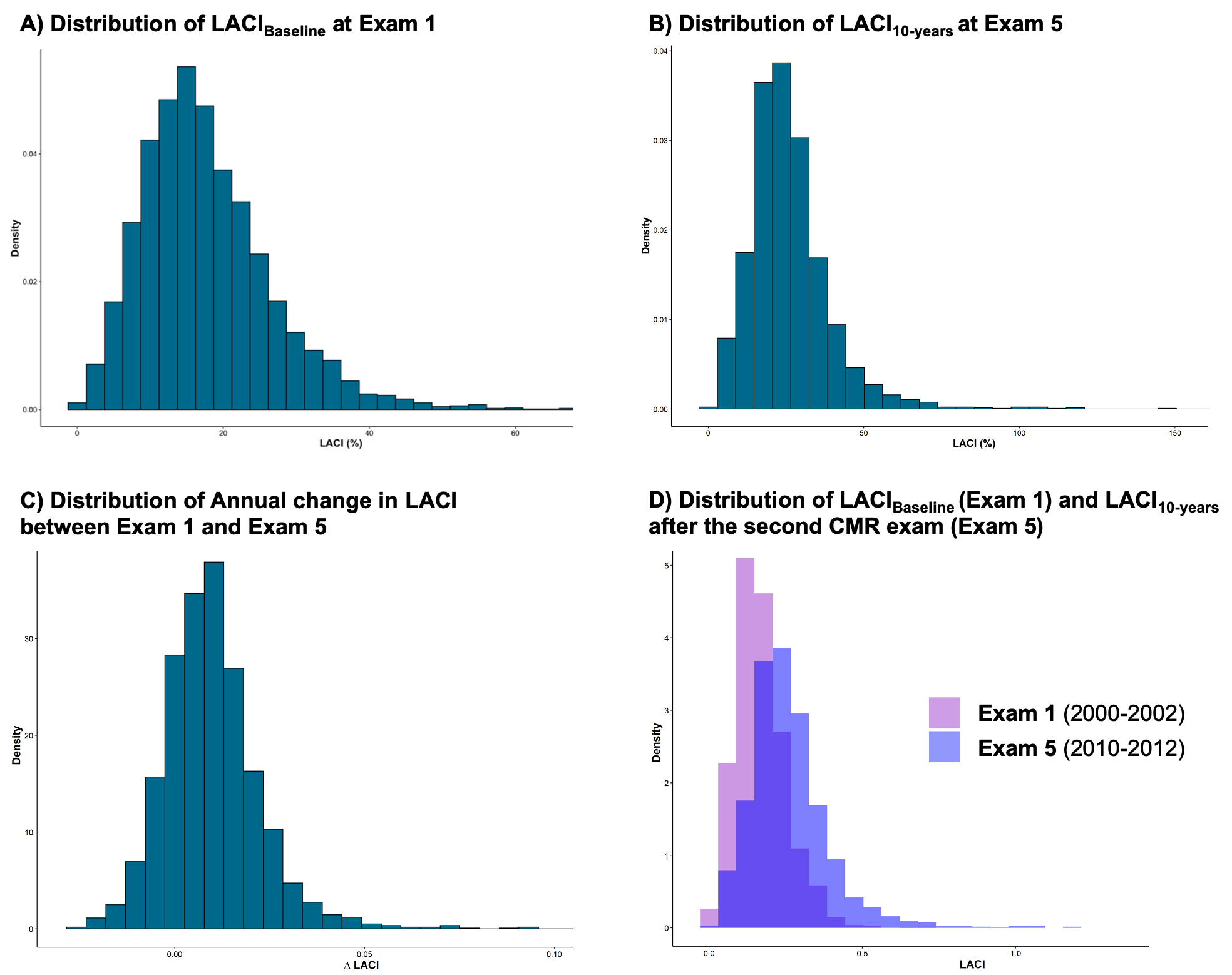
**

**SUPPLEMENTARY FILE 4:**

**Annual change in LACI, LA and LV variables between Exam 1 and Exam 5.**

| **Parameters** | **All population**  **(n=2,250)** | **No HF**  **(n=2,200)** | **HF**  **(n=50)** | **p-value** |
| --- | --- | --- | --- | --- |
| **∆**LACI (%/year) | 1.3 ± 1.0 | 1.3 ± 1.0 | 2.4 ± 2.0 | **<0.001** |
| **∆**LAVI_min_ (mL/m^2^/year) | 0.49 ± 0.83 | 0.47 ± 0.81 | 1.29 ± 1.28 | **<0.001** |
| **∆**LAVI_max_ (mL/m^2^/year) | 0.57 ± 1.16 | 0.55 ± 1.15 | 1.16 ± 1.34 | **<0.001** |
| **∆**Peak LA strain (%/year) | -0.59 ± 1.65 | -0.57 ± 1.63 | -1.35 ± 2.03 | **0.010** |
| **∆**LV EDVi (mL/m^2^/year) | -0.68 ± 1.21 | -0.68 ± 1.20 | -0.62 ± 1.43 | 0.726 |
| **∆**LVEF (%/year) | -0.07 ± 0.78 | -0.06 ± 0.78 | -0.27 ± 0.93 | 0.117 |
| **∆**LV mass index (g/m^2^/year) | 0.03 ± 0.02 | 0.07 ± 0.05 | 1.13 ± 1.02 | **<0.001** |
| **∆**LV MVR | 0.01 ± 0.02 | 0.01 ± 0.02 | 0.02 ± 0.03 | 0.091 |
| **∆**LVGFI (%/year) | -0.31 ± 0.75 | -0.30 ± 0.74 | -0.53 ± 0.83 | 0.060 |
| **∆**Framingham CVD risk (%/year) | 0.32 ± 0.67 | 0.32 ± 0.67 | 0.25 ± 0.69 | 0.474 |

Abbreviations: CVD: cardiovascular disease; HF: heart failure; LA: left atrium; LACI: left atrioventricular coupling index; LAVI: left atrium volume indexed; EDVi: end-diastolic volume indexed; LV: left ventricle; LVEF: left ventricle ejection fraction; LVGFI: LV global function index; LVMVR: LV mass/LV volume.

**SUPPLEMENTARY FILE 5:**

**Relationship between the LA end-diastolic volume and LV end-diastolic volume used to define the LACI_Baseline_ measured at Exam 1 (A) LACI_10-years_ measured at Exam 5 (B).**


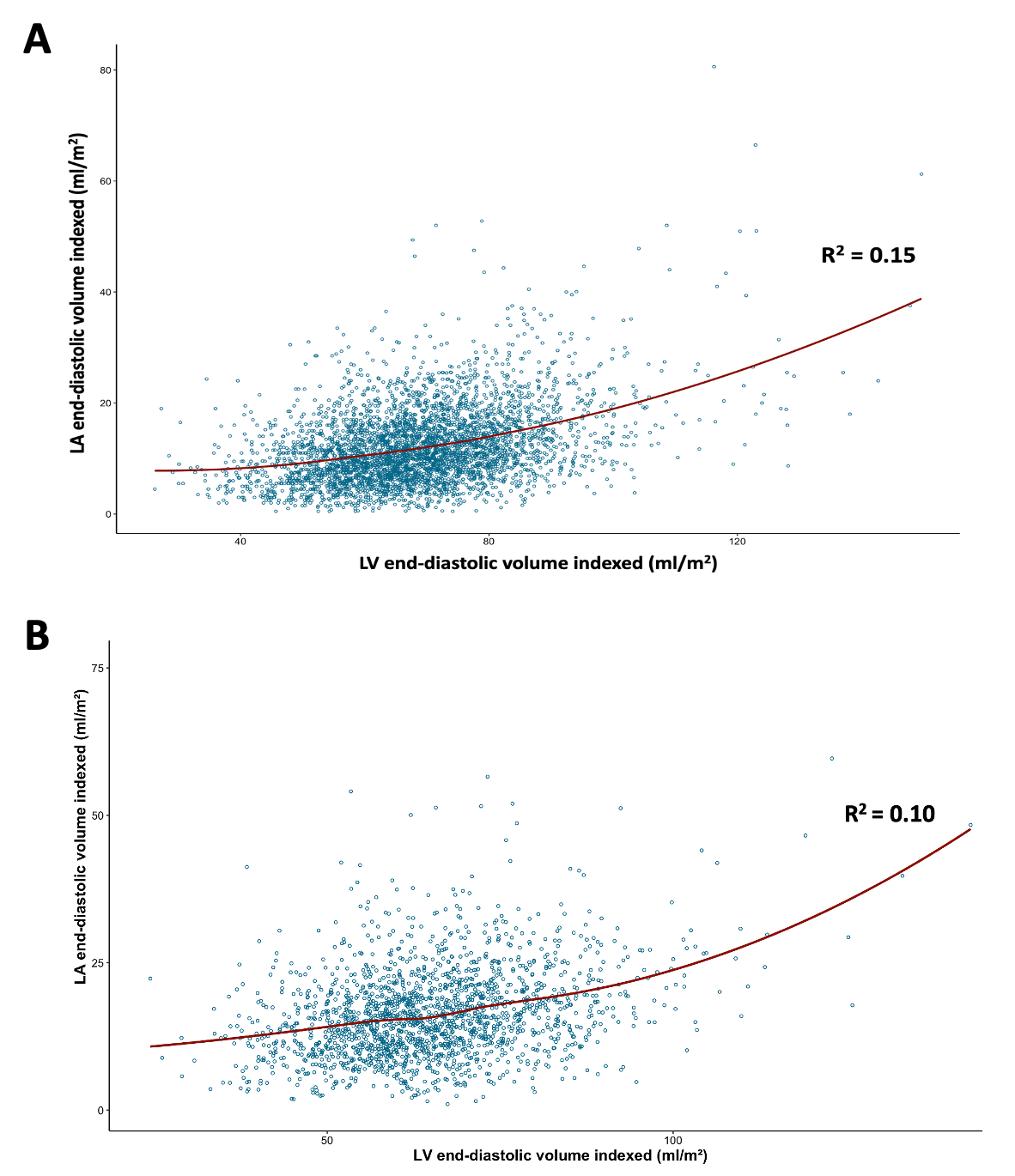


**SUPPLEMENTARY FILE 6:**

**LACI and Annual change in LACI regarding the participant’s Sex.**

| Parameters | **Women**  **(n=1200)** | **Males**  **(n=1050)** | **p-value** |
| --- | --- | --- | --- |
| LACI_Baseline_ at Exam 1, % | 16.7 ± 8.2 | 16.8 ± 7.6 | 0.661 |
| LACI_10-years_ at Exam 5, % | 26.3 ± 12.0 | 24.7 ± 11.2 | **0.010** |
| Annual change in LACI, %/year | 1.03 ± 1.10 | 0.83 ± 1.00 | **<0.001** |

**SUPPLEMENTARY FILE 7:**

**Determination of the optimal cut-off to transform the LACI into a binary variable with the best predictive value for incident heart failure.**

The survival tree method for censored data was used to identify the cut-off to transform the annual change in LACI into a binary variable with the best predictive value. The best LACI cut-off to predict incident HF was 30%.

**
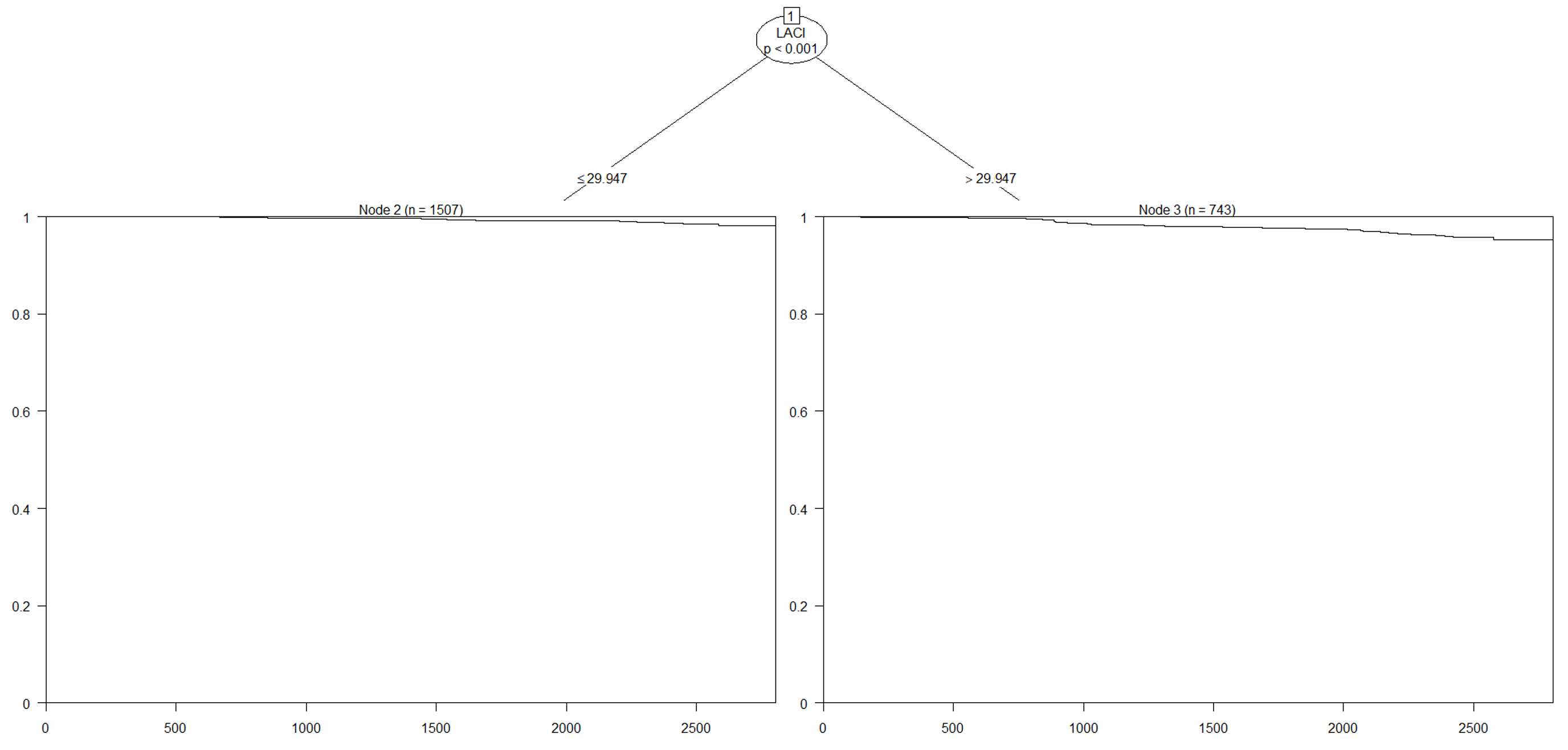
**

**SUPPLEMENTARY FILE 8:**

**Determination of the optimal cut-off to transform the annual change in LACI between Exam 1 and Exam 5 into a binary variable with the best predictive value for incident heart failure.**

The survival tree method for censored data was used to identify the cut-off to transform the annual change in LACI into a binary variable with the best predictive value. The best annual change in LACI cut-off to predict incident heart failure was 1.5%.


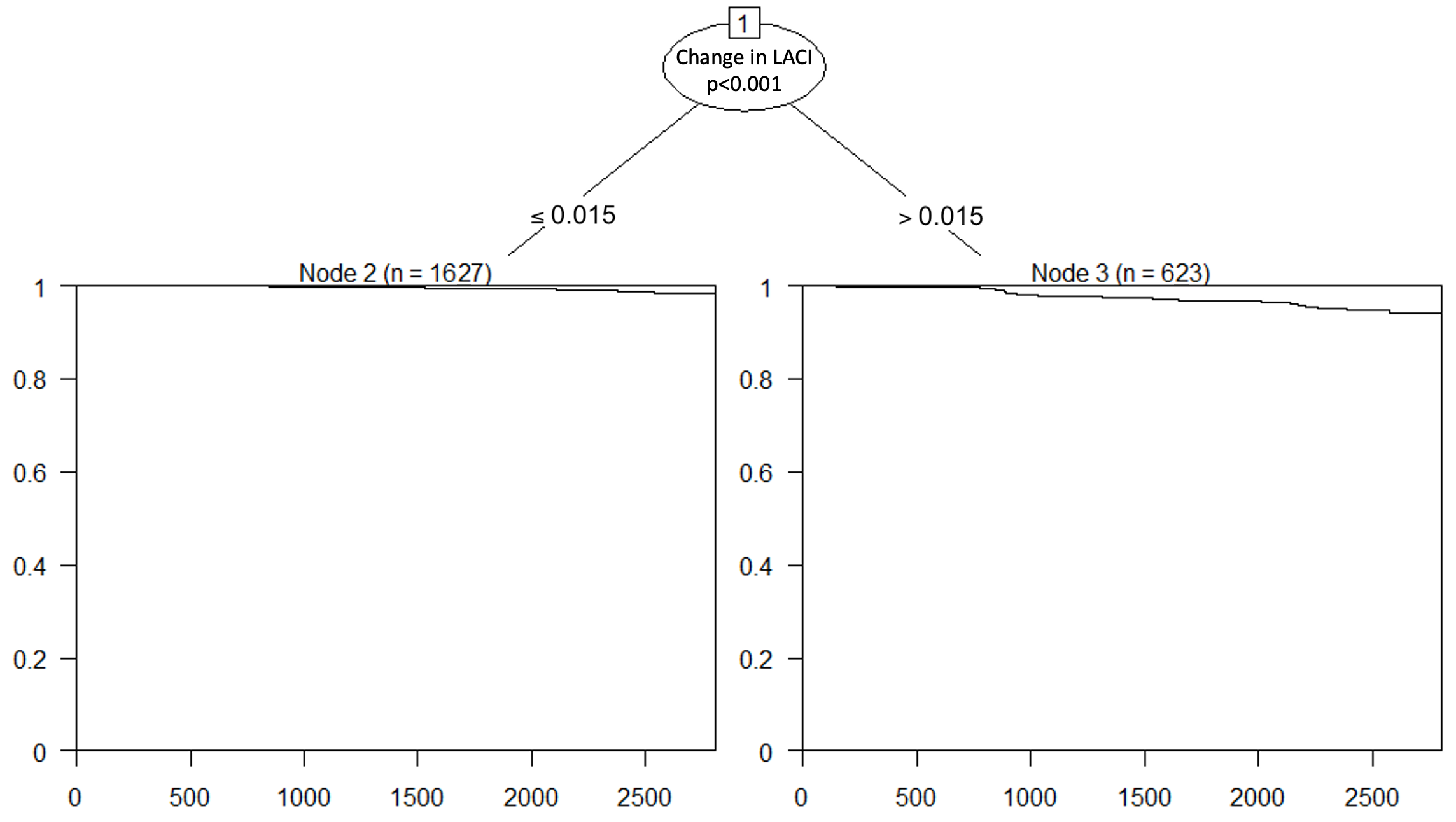

Supplement: Supplementary file 1 [file Data_Sheet_1.docx]
