# Supplementary material for: Left Atrioventricular Coupling Index to Predict Incident Heart Failure: The Multi-Ethnic Study of Atherosclerosis
Source: Front Cardiovasc Med. 2021 Sep 1;8:704611. doi: 10.3389/fcvm.2021.704611 (PMC8442844; doi:10.3389/fcvm.2021.704611)
Supplement: Supplementary file 2 [file Table_1.DOCX]

**TABLES**

**Table 1. Population characteristics of participants at baseline and at second examination (n=2,250)**

| **Parameters** | **Baseline (Exam 1)**  **(n=2,250)** | **Second study (Exam 5), 9.6 ± 0.6 years after baseline** | | |  |
| --- | --- | --- | --- | --- | --- |
|  |  | **No HF (n=2,200)** | **HF (n=50)** | **p-value** |  |
| Age, years | 59.3 ± 9.3 | 68.6 ± 9.1 | 76.0 ± 8.9 | **<0.001** |  |
| Male, n (%) | 1050 (46.7) | 1026 (46.6) | 24 (48.0) | 0.962 |  |
| Ethnicity (Ca/Ch/AA/Hi), % | 43/122/24/21 | 43/12/24/21 | 25/1/12/12 | 0.171 |  |
| Hypertension, n (%) | 840 (37.3) | 1226 (55.7) | 45 (90.0) | **<0.001** |  |
| Systolic blood pressure, mmHg | 123 ± 20 | 123 ± 20 | 135 ± 24 | **0.001** |  |
| Diastolic blood pressure, mmHg | 72 ± 10 | 68 ± 10 | 69 ± 11 | 0.839 |  |
| Hypertension medication, n (%) | 701 (31.2) | 1130 (51.4) | 40 (80.0) | **<0.001** |  |
| Body mass index, kg/m^2^ | 27.8 ± 5.0 | 28.1 ± 5.2 | 28.7 ± 5.3 | 0.399 |  |
| Glycemic status, n (%) |  |  |  | **0.021** |  |
| Normal | 1781 (79.2) | 1381 (62.8) | 23 (46.0) |  |  |
| Impaired fasting glucose | 254 (11.3) | 443 (20.1) | 13 (26.0) |  |  |
| Diabetes mellitus | 215 (9.6) | 376 (15.7) | 14 (28.0) |  |  |
| Smoking status, n (%) |  |  |  | **0.017** |  |
| Never | 1178 (52.4) | 1023 (46.5) | 14 (28.0) |  |  |
| Former | 817 (36.3) | 1013 (46.0) | 33 (66.0) |  |  |
| Current | 255 (11.3) | 164 (7.5) | 3 (6.0) |  |  |
| LDL cholesterol, mg/dl | 118 ± 31 | 107 ± 32 | 91 ± 32 | **0.001** |  |
| HDL cholesterol, mg/dl | 51 ± 15 | 56 ± 16 | 56.4 ± 18 | 0.877 |  |
| Lipid-lowering medication, n (%) | 331 (14.7) | 811 (36.9) | 24 (48.0) | 0.143 |  |
| NT-proBNP, pg/ml | 73.6 ± 108.2 | 117.7 ± 122.0 | 463.4 ± 232.2 | **<0.001** |  |
| Framingham CVD risk, % | 12.3 ± 8.9 | 15.2 ± 9.0 | 20.6 ± 8.5 | **<0.001** |  |
| Heart rate, bpm | 62 ± 8.9 | 64.2 ± 10.4 | 66.8 ± 10.4 | 0.092 |  |
| LA parameters |  |  |  |  |  |
| LAVI_min_, ml/m^2^ | 11.9 ± 6.2 | 16.3 ± 8.3 | 26.1 ± 16.3 | **<0.001** |  |
| LAVI_max_, ml/m^2^ | 30.0 ± 9.4 | 35.1 ± 11.2 | 43.9 ± 16.4 | **<0.001** |  |
| Peak LA reservoir strain, % | 37.0 ± 11.0 | 31.7 ± 13.7 | 23.9 ± 16.5 | **0.002** |  |
| LV parameters |  |  |  |  |  |
| LV EDVi, ml/m^2^ | 70.9 ± 12.1 | 64.4 ± 13.2 | 67.0 ± 17.3 | 0.295 |  |
| LVEF, % | 62.6 ± 5.7 | 62.1 ± 7.1 | 59.1 ± 9.16 | **0.027** |  |
| LV mass index, g/m^2^ | 65.0 ± 11.6 | 65.7 ± 13.4 | 76.2 ± 16.3 | **<0.001** |  |
| LV MVR, g/ml | 0.93 ± 0.17 | 1.04 ± 0.22 | 1.20 ± 0.35 | **0.005** |  |
| LVGFI, % | 40.4 ± 6.1 | 37.6 ± 6.7 | 33.0 ± 7.4 | **<0.001** |  |
| LACI, % | 17.0 ± 8.0 | 26.1 ± 10.2 | 41.2 ± 12.1 | **<0.001** |  |
|  |  |  |  |  |  |

Abbreviations: AA: African American; Ca: Caucasian; Ch: Chinese American; Hi: Hispanic; NT-proBNP: N-terminal prohormone of brain natriuretic peptide; CVD: cardiovascular disease; HDL: high-density lipoprotein; HF: heart failure; LA: left atrium; LACI: left atrioventricular coupling index; LAVI: left atrium volume indexed; LDL: low-density lipoprotein; EDVi: end-diastolic volume indexed; LV: left ventricle; LVEF: left ventricle ejection fraction; LVGFI: LV global function index; LVMVR: LV mass/LV volume.

**Table 2. Univariable and multivariable analysis of incident HF according to LACI and other LA or LV parameters after 10 years**

|  | **Univariable analysis** | |  | **Model 1***  **HF risk factors** | |  |
| --- | --- | --- | --- | --- | --- | --- |
|  | **Hazard Ratio**  **(95% CI)** | **p value** |  | **Hazard Ratio**  **(95% CI)** | **p value** |  |
|  |  |  |  |  |  |  |
| LACI_10-years_ ^†^ | 1.69 (1.50-1.90) | **<0.001** |  | 1.44 (1.25-1.66) | **<0.001** |  |
| LACI_10-years_ cut-off >30% ^‡^ | 4.47 (2.57-7.79) | **<0.001** |  | 2.05 (1.14-3.68) | **0.011** |  |
| LAVI_min_ | 1.67 (1.47-1.88) | **<0.001** |  | 1.40 (1.28-1.68) | **<0.001** |  |
| LAVI_max_ | 1.64 (1.36-1.98) | **<0.001** |  | 1.35 (1.08-1.69) | **0.023** |  |
| Peak LA reservoir strain | 0.75 (0.58-0.88) | **0.003** |  | 0.79 (0.65-0.92) | **0.012** |  |
| LV EDVi | 1.20 (0.92-1.57) | 0.174 |  | 0.95 (0.78-1.16) | 0.619 |  |
| LVEF | 0.65 (0.50-0.85) | **0.002** |  | 0.70 (0.55-0.89) | **0.008** |  |
| LV mass index | 1.58 (1.30-1.97) | **<0.001** |  | 1.22 (1.03-1.52) | **0.032** |  |
| LV MVR | 1.64 (1.34-2.02) | **<0.001** |  | 1.37 (1.07-1.74) | **0.016** |  |
| LVGFI | 0.49 (0.37-0.65) | **<0.001** |  | 0.54 (0.40-0.74) | **<0.001** |  |
| Framingham CVD risk | 1.84 (1.39-2.43) | **<0.001** |  | 1.00 (0.64-1.57) | 0.984 |  |

* Multivariable **model 1** (HF risk model) included: age, gender, race, body mass index, hypertension, diabetes, smoking status dyslipidemia and NT-proBNP.

Of note, each line of this table corresponds to the addition one by one of the LV or LA parameters to the model 1.

^†^ LACI_10-years_ used as continuous variable

^‡^ LACI_10-years_ used as binary variable defined by a cut-off >30%.

All LV parameters, LA parameters and LACI values were normalized according to the following formula: (parameter–mean value)/standard deviation.

*Abbreviations*: CI: confidence interval; CVD: cardiovascular disease; EDVi: end-diastolic volume indexed; EF: emptying fractions; HF: heart failure; Indexed volumes: maximum (VImax), minimum (VImin); LA: left atrial; LACI: left atrioventricular coupling index; LAVI: left atrium volume indexed; LV: left ventricle; LVEF: left ventricle ejection fraction; LVGFI: LV global function index; MVR: mass-to-volume ratio.

**Table 3. Bivariable and multivariable analysis of incident HF according to Annual change in LACI and Annual change in other LA or LV parameters.**

|  | **Bivariable analysis*** | |  | **Model 1**^†^  **HF risk factors** | |  | **Model 2**^‡^  **Model 1 + Baseline LA/LV variables** | |  |
| --- | --- | --- | --- | --- | --- | --- | --- | --- | --- |
|  | **Hazard Ratio**  **(95% CI)** | **p value** |  | **Hazard Ratio**  **(95% CI)** | **p value** |  | **Hazard Ratio**  **(95% CI)** | **p value** |  |
|  |  |  |  |  |  |  |  |  |  |
| **∆**LACI^§^ | 1.77 (1.49-2.09) | **<0.001** |  | 1.56 (1.32-1.85) | **<0.001** |  | 1.55 (1.30-1.85) | **<0.001** |  |
| **∆**LACI cut-off>1.5%/year^\|\|^ | 3.74 (2.14-6.55) | **<0.001** |  | 2.53 (1.44-4.46) | **<0.001** |  | 2.68 (1.51-4.75) | **<0.001** |  |
| **∆**LAVI_min_ | 1.69 (1.47-1.93) | **<0.001** |  | 1.50 (1.25-1.80) | **<0.001** |  | 1.48 (1.22-1.79) | **<0.001** |  |
| **∆**LAVI_max_ | 1.52 (1.31-2.02) | **<0.001** |  | 1.45 (1.11-1.90) | **<0.001** |  | 1.52 (0.97-1.62) | 0.064 |  |
| ∆Peak LA reservoir strain | 0.72 (0.56-0.87) | **0.002** |  | 0.88 (0.62-1.04) | 0.078 |  | 0.70 (0.52-0.85) | **0.019** |  |
| **∆**LV EDVi | 1.17 (0.87-1.58) | 0.293 |  | 1.15 (0.89-1.47) | 0.279 |  | 1.14 (0.87-1.46) | 0.291 |  |
| **∆**LVEF | 0.68 (0.51-0.91) | **0.009** |  | 0.78 (0.60-1.01) | 0.055 |  | 0.67 (0.50-0.88) | **0.004** |  |
| **∆**LV mass index | 1.59 (1.39-2.10) | **<0.001** |  | 1.27 (0.99-1.61) | 0.065 |  | 1.51 (1.26-1.82) | **<0.001** |  |
| **∆**LV MVR | 1.48 (1.19-1.85) | **<0.001** |  | 1.24 (0.98-1.57) | 0.071 |  | 1.32 (1.04-1.67) | **0.020** |  |
| **∆**LVGFI | 0.51 (0.37-0.70) | **<0.001** |  | 0.77 (0.59-1.00) | 0.051 |  | 0.75 (0.56-1.05) | 0.065 |  |
| **∆**Framingham CVD risk | 1.20 (0.89-1.60) | 0.228 |  | 1.01 (0.79-1.29) | 0.920 |  | 1.02 (0.74-1.40) | 0.912 |  |

* Bivariable model included both the annual change in the variable and the value of the variable measured at baseline.

† Multivariable **model 1** (HF risk model) included: age, gender, race, body mass index, hypertension, diabetes, smoking status dyslipidemia and NT-proBNP.

^‡^ Multivariable **model 2** included: model 1 + baseline value measured at Exam 1 for each LA or LV parameters.

Of note, each line of this table corresponds to the addition one by one of the changes in LV or LA parameters to the models 1 or 2.

^§^ **∆**LACI used as continuous variable

^||^ **∆**LACI used as binary variable defined by a cut-off>1.5%/year.

All variables values were expressed per 1-SD/year and normalized according to the following formula: (Variable measured – mean value)/standard deviation.

*Abbreviations:* **∆**: Annual change; CI: confidence interval; CVD: cardiovascular disease; EDVi: end-diastolic volume indexed; EF: emptying fractions; HF: heart failure; Indexed volumes: maximum (VImax), minimum (VImin); LA: left atrial; LACI: left atrioventricular coupling index; LV: left ventricle; LVEF: left ventricle ejection fraction; LVGFI: LV global function index; MVR: mass-to-volume ratio.

**Table 4. Discrimination and reclassification associated with LACI to different LA and LV parameters at 10-years of follow-up to predict incident HF.**

|  | **Incident HF** | | |  |
| --- | --- | --- | --- | --- |
|  | **C-index**  **(95%CI)** | **NRI**  **(95%CI)** | **IDI**  **(95%CI)** |  |
|  |  |  |  |  |
| Model 1^*^ (HF risk factors) | 0.77 (0.73–0.82) | Reference | Reference |  |
|  |  |  |  |  |
| Model 1 + LACI_10-years_ ^†^ | 0.81 (0.74–0.87) | 0.411 (0.042–0.780) | 0.043 (0.016–0.106) |  |
| Model 1 + LACI_10-years_ cut-off > 30% ^‡^ | 0.80 (0.73–0.86) | 0.607 (0.063–0.843) | 0.039 (0.011–0.107) |  |
| Model 1 + LAVI_min_ | 0.80 (0.73–0.86) | 0.201 (-0.219–0.486) | 0.038 (0.010–0.104) |  |
| Model 1 + LAVI_max_ | 0.78 (0.74–0.82) | 0.328 (0.050-0.573) | 0.015 (0.004–0.041) |  |
| Model 1 + Peak LA reservoir strain | 0.79 (0.73–0.85) | 0.312 (0.047-0.599) | 0.017 (0.006–0.044) |  |
| Model 1 + LV EDVi | 0.77 (0.73–0.82) | 0.075 (-0.222–0.372) | 0.000 (-0.001 - 0.010) |  |
| Model 1 + LVEF | 0.80 (0.74–0.86) | 0.369 (0.158–0.580) | 0.039 (0.010–0.109) |  |
| Model 1 + LV mass index | 0.79 (0.72–0.85) | 0.248 (0.137–0.398) | 0.018 (0.009–0.067) |  |
| Model 1 + LV MVR | 0.79 (0.73–0.85) | 0.259 (0.143–0.402) | 0.020 (0.012–0.069) |  |
| Model 1 + LVGFI | 0.80 (0.74–0.85) | 0.382 (0.157–0.607) | 0.031 (0.015–0.085) |  |
| Model 1 + Framingham CVD risk | 0.77 (0.73–0.82) | 0.065 (-0.192–0.337) | 0.001 (-0.001–0.012) |  |

* Multivariable **model 1** (HF risk model) included: age, gender, race, body mass index, hypertension, diabetes, smoking status, dyslipidemia and NT-proBNP.

^†^ LACI_10-years_ used as continuous variable

^‡^ LACI_10-years_ used as binary variable defined by a cut-off > 30%.

All LV parameter, LA parameter and LACI values were normalized according to the following formula: (parameter–mean value)/standard deviation.

For each model, discrimination and reclassification were based on net reclassification improvement (NRI) and integrated discrimination improvement (IDI). Results are for 7-year follow-up.

*Abbreviations:* CI: confidence interval; CVD: cardiovascular disease; EDVi: end-diastolic volume indexed; EF: emptying fractions; HF: heart failure; Indexed volumes: maximum (VImax), minimum (VImin); LA: left atrial; LACI: left atrioventricular coupling index; LV: left ventricle; LVEF: left ventricle ejection fraction; MVR: mass-to-volume ratio.

**Table 5. Discrimination and reclassification associated with Annual change in LACI to change in different LA and LV parameters to predict incident HF.**

|  | **Incident HF** | | |  |
| --- | --- | --- | --- | --- |
|  | **C-index**  **(95%CI)** | **NRI**  **(95%CI)** | **IDI**  **(95%CI)** |  |
|  |  |  |  |  |
| Model 1^*^ (HF risk factors) | 0.77 (0.73-0.82) | Reference | Reference |  |
|  |  |  |  |  |
| Model 2^†^ + **∆**LACI ^‡^ | 0.82 (0.76-0.89) | 0.491 (0.048-0.934) | 0.058 (0.028-0.096) |  |
| Model 2^†^ + **∆**LACI cut-off>1.5%/year ^§^ | 0.81 (0.75-0.87) | 0.536 (0.050-0.998) | 0.045 (0.024-0.083) |  |
| Model 2^†^ + **∆**LAVI_min_ | 0.80 (0.75-0.85) | 0.455 (0.003-0.907) | 0.031 (0.008-0.076) |  |
| Model 2^†^ + **∆**LAVI_max_ | 0.79 (0.74-0.83) | 0.270 (-0.010-0.482) | 0.019 (0.002–0.072) |  |
| Model 2 + **∆**Peak LA reservoir strain | 0.77 (0.73-0.82) | 0.009 (-0.178-0.281) | 0 (-0.002-0.007) |  |
| Model 2^†^ + **∆**LV EDVi | 0.77 (0.73-0.82) | -0.013 (-0.172-0.198) | 0 (-0.002-0.009) |  |
| Model 2^†^ + **∆**LVEF | 0.77 (0.73-0.82) | 0.010 (-0.182-0.278) | 0 (-0.001-0.008) |  |
| Model 2^†^ + **∆**LV mass index | 0.80 (0.75-0.84) | 0.428 (0.002-0.876) | 0.030 (0.007-0.075) |  |
| Model 2^†^ + **∆**LV MVR | 0.79 (0.73-0.85) | 0.251 (-0.030-0.532) | 0.016 (0.002–0.053) |  |
| Model 2^†^ + **∆**LVGFI | 0.80 (0.74-0.86) | 0.466 (0.006-0.926) | 0.033 (0.011-0.080) |  |
| Model 2^†^ + **∆** Framingham CVD risk | 0.77 (0.73-0.82) | -0.052 (-0.246-0.262) | 0 (-0.001-0.008) |  |

^*^ Multivariable **model 1** (HF risk model) included: age, gender, race, body mass index, hypertension, diabetes, smoking status dyslipidemia and NT-proBNP.

^†^ Multivariable **model 2** included: model 1 + baseline value measured at Exam 1 for each LA or LV parameters.

^‡^ **∆**LACI used as continuous variable

^§^ **∆**LACI used as binary variable defined by a cut-off>1.5%/year.

All variables values were expressed per 1-SD/year and normalized according to the following formula: (Variable measured – mean value)/standard deviation.

For each model, discrimination and reclassification were based on net reclassification improvement (NRI) and integrated discrimination improvement (IDI). Results are for 7-year follow-up.

*Abbreviations:* **∆**: Annual change; CI: confidence interval; CVD: cardiovascular disease; EDVi: end-diastolic volume indexed; EF: emptying fractions; Indexed volumes: maximum (VImax), minimum (VImin); LA: left atrial; LACI: left atrioventricular coupling index; LV: left ventricle; LVEF: left ventricle ejection fraction; MVR: mass-to-volume ratio.
